# Patterns of care and treatment outcomes of patients with astroblastoma: a National Cancer Database analysis

**DOI:** 10.2217/cns-2017-0038

**Published:** 2018-04-30

**Authors:** Emily C Merfeld, Sonika Dahiya, Stephanie M Perkins

**Affiliations:** 1 Department of Radiation Oncology, Washington University School of Medicine in St. Louis, St. Louis, MO 63110 USA; 2 Department of Pathology & Immunology, Washington University School of Medicine in St. Louis, St. Louis, MO 63110 USA

**Keywords:** astroblastoma, brain tumor, neuro-oncology, pediatric oncology, rare tumor

## Abstract

**Aim:**

To evaluate the use of chemotherapy and radiation, and their outcomes for patients with astroblastoma.

**Patients & methods:**

This is a retrospective review of patients extracted from the National Cancer Database. We investigated overall survival (OS) using Kaplan–Meier curves. Cox proportional hazards models were used to correlate OS with risk variables and treatments.

**Results:**

OS at 5 years was 79.5%. Patients with high-grade tumors were more likely to receive chemotherapy and radiation. Patients with high-grade astroblastoma who did not receive adjuvant radiation had poor survival.

**Conclusion:**

Patients with astroblastoma should be treated with curative intent. Radiation is likely beneficial in high-grade astroblastoma. The exact role of radiation and chemotherapy following surgical resection warrant further investigation.

**Summary points**

- Astroblastoma displays unpredictable clinical behavior, and limited data exists on its management.
- Gross total resection of astroblastoma is associated with increased survival, but the role of adjuvant chemotherapy and radiation therapy are controversial.
- Patients with astroblastoma should be treated with curative intent, as the majority of patients become long-term survivors.
- Patients with high-grade tumors are more likely to receive chemotherapy and radiation.
- Larger studies with central pathologic review are necessary to define the association between pathohistological features and prognosis.
- The use of adjuvant chemotherapy in astroblastoma cannot be supported.
- Patients with high-grade astroblastoma who do not receive adjuvant radiation have poor survival. Radiation is likely beneficial for patients with high-grade astroblastoma.
- The exact role of radiation and chemotherapy following surgical resection of astroblastoma require further investigation.

Astroblastoma is a rare brain tumor with incidence between 0.45 and 2.8% of gliomas [1]. The characteristic histopathological feature of astroblastoma is the astroblastic pseudorosette. The tumor is most often observed in pediatrics,  adolescents and young adults (AYAs) but is also identified in adults [2]. Genetic differences in the tumors of younger versus older patients have been suggested [3].

Astroblastoma displays unpredictable clinical behavior. Two histopathological categories of the tumor are distinguished in the literature, low and high grade. Low versus high-grade pathology have been associated with corresponding prognoses in small case series [4–8], though reviews including larger numbers of patients dispute this correlation [9]. Numerous case reports present discordant tumor grade and behavior, transformation of low- to high-grade tumors and tumor recurrence with a different histologic grade [5,9–12].

There is no level 1 or 2 data on the treatment of astroblastoma, and practices are inconsistent. Radiographically and microscopically, astroblastomas are well circumscribed and noninfiltrative [13]. Gross total resection is associated with increased survival [2,3]. The role of adjuvant therapy remains in question. Single institution reviews offer support for radiation therapy (RT) [8,15], and one literature review of 20 patients with high-grade tumors noted superior survival rates in patients who received surgery and adjuvant RT [12]. However, the two largest analyses of astroblastoma patients failed to show a benefit of RT [2,3]. Notably, the largest report of 239 astroblastoma patients by Ahmed *et al*. lacked information on tumor grade, type and dose of RT and chemotherapy [3].

The objective of the current study is to evaluate the recent pattern of care regarding the use of chemoradiation and their outcomes for pediatric, adolescent and young adult patients with astroblastoma in the National Cancer Database (NCDB) after 2004.

## Patients & methods

This is a retrospective review of patients with astroblastoma extracted from the NCDB. The NCDB is jointly sponsored by the American College of Surgeons and the American Cancer Society and serves as a database on clinical oncology. The database represents 70% of newly diagnosed malignancies in USA. Hospitals accredited by the Commission on Cancer collect additional details on RT and chemotherapy that are not captured by the Surveillance, Epidemiology and End Results registry. Deidentified data for patients aged 0–39 with histologically confirmed astroblastoma (ICD-0–3 histology code 6430) diagnosed from 2004 to 2012 were obtained from the NCDB participant user file. Patients of age 0–39 were chosen for evaluation to ensure analysis of a genetically and pathologically uniform group of astroblastoma, given suggestions of genetic difference in the tumors of older patients [3]. Institutional Review Board approval was waived for this study, as data from the NCDB is deidentified for patient and institution.

Patient's demographic information was collected including age, gender and race. Tumor factors including location, size and grade were also collected. Patients of age 0–14 were categorized as pediatrics, and patients of age 15–39 were categorized as AYA. Grades 1 and 2 were categorized as low grade. Grades 3 and 4 were categorized as high grade. Treatment data collected included whether patients had surgery, details of RT including dose and technique and chemotherapy including single versus multiagent therapy. The survival time was calculated as the number of years from diagnosis to the NCDB date of death.

To investigate overall survival (OS), Kaplan–Meier curves were generated for the patient cohort, and subgroups including tumor grade, tumor size and age were compared using the log-rank test. Univariate analysis was performed using cox proportional hazards models to estimate hazard ratios for risk variables and treatments, including age, gender, grade, tumor size, RT, RT dose and chemotherapy. The difference in treatment patterns observed between high and low-grade tumors prompted subanalysis of patients with high-grade tumors. Kaplan–Meier curves were generated and the log-rank test was used to compare treatment groups, including chemotherapy and RT. Figures were generated using IBM SPSS Statistics, version 25 (IBM, NY, USA).

## Results

### Patient characteristics

Within the NCDB, we identified 63 patients diagnosed with astroblastoma between 2004 and 2012. Demographic, tumor and treatment information are shown in Table 1. Median age at the time of diagnosis was 16 years. Median follow-up was 5.5 years. The cohort was made up of 49 females (76.6%). Tumor location was known for 55 (87%) patients. Of these, 50 (90.1%) patients were in a supratentorial location. Assigned histopathological grade was known for 38 (60%) patients. Of these, 18 (47.4%) patients were low grade, while 20 (52.6%) patients were high grade. Median tumor size was 4.4 cm (range: 1.1–10.7).

**Table 1.** **Patient demographics, tumor information and treatment information of cohort.**

**Variable**	**Categories**	**Number (%)**
Gender	Female	49 (76.6)

	Male	14 (21.9)

Age	<5	5 (7.8)

	5–14	24 (37.5)

	15–29	22 (34.4)

	≥30	12 (18.8)

Race	White	51 (82.2)

	Black	7 (11.3)

	Other	4 (6.5)

Tumor location	Frontal lobe	24 (43.6)

	Temporal lobe	9 (16.4)

	Parietal lobe	12 (21.8)

	Occipital lobe	5 (9.1)

	Brainstem	2 (3.6)

	Intraventricular	3 (5.5)

Size	<3 cm	10 (21.3)

	3–5 cm	19 (40.4)

	>5 cm	18 (38.3)

Grade	1	10 (26.3)

	2	8 (21.1)

	3	3 (7.9)

	4	17 (44.7)

Surgery	Yes	59 (98.3)

	No	1 (1.7)

RT	Yes	26 (43.3)

	No	34 (56.7)

RT dose	39.6–54 Gy	8 (33.3)

	54 Gy	7 (29.2)

	54–69.4 Gy	9 (37.5)

Chemotherapy	Yes	20 (31.3)

	No	43 (67.2)

RT: Radiation therapy.

### Treatment

**Figure 1.**
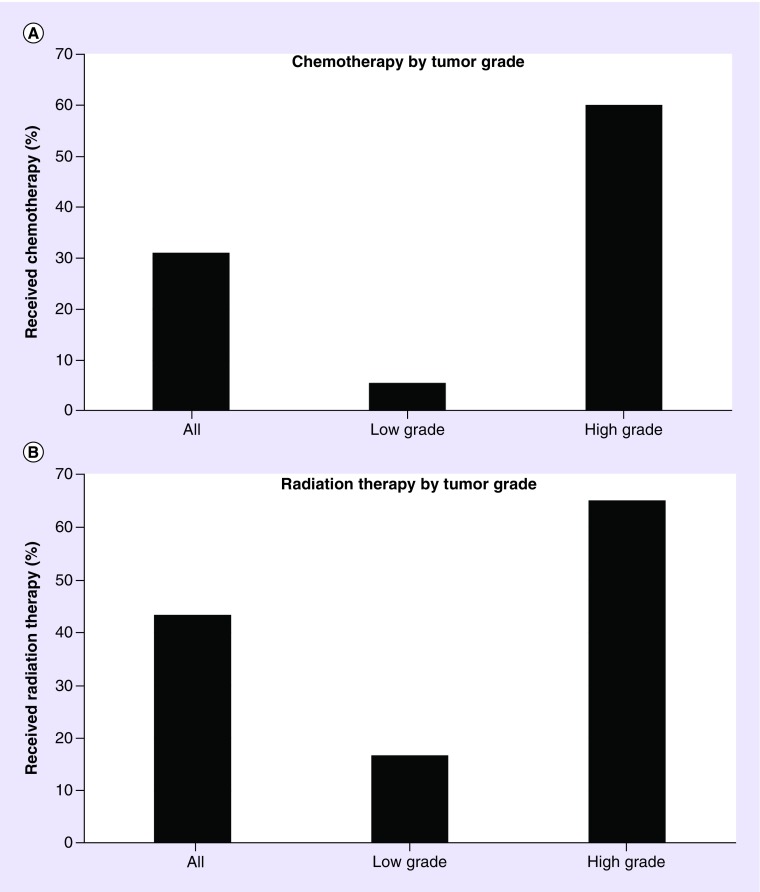
**Patterns of care by tumor grade.** **(A)** Treatment with chemotherapy according to tumor grade. **(B)** Treatment with radiation therapy according to tumor grade.

All but one patient was treated with surgical resection. Extent of resection was known for only 33% of patients. This variable was thus excluded from analysis. A total of 20 (31%) patients received chemotherapy. Of patients with high-grade tumors, 60% received chemotherapy. Of patients with low-grade tumors, 5.6% received chemotherapy (Figure 1A). Six (30%) of these patients received multiagent chemotherapy, while 14 (70%) patients received single-agent chemotherapy.

A total of 26 (43%) patients received RT. Of patients with high-grade tumors, 65% were treated with RT. Of patients with low-grade tumors, 17% were treated with RT (Figure 1B). A total of 15 (57.7%) patients received intensity-modulated RT or 3D conformal RT; no patients were reported to have received proton therapy. The median dose of radiation during the first course of treatment was 54 Gy. Nine patients received a boost, with a median dose of 45 Gy prior to the boost and a median boost dose of 14 Gy.

### Outcomes

**Figure 2.**
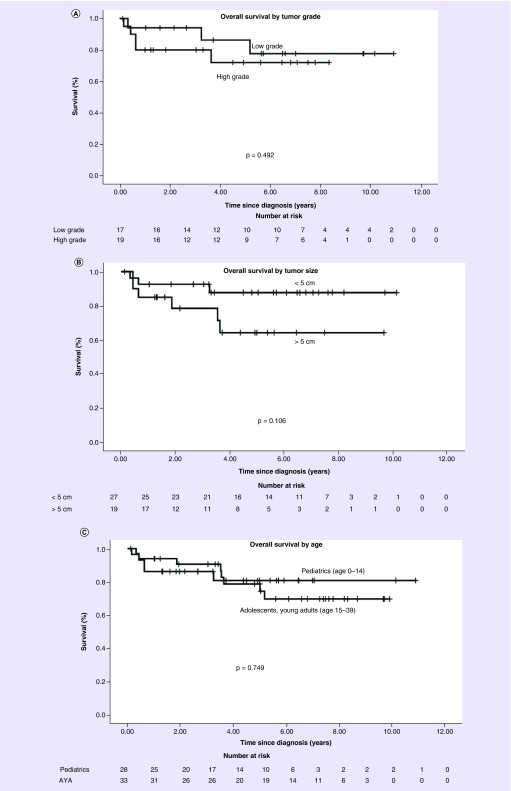
**Overall survival by tumor and patient characteristics.** **(A)** Overall survival in low versus high grade astroblastoma. **(B)** Overall survival in tumors <5 cm versus >5 cm. **(C)** Overall survival in pediatric patients (age 0-14) versus adolescents and young adults (ages 15-39). AYA: Adolescents and young adult.

OS at 5 years was 79.5%. Patients with low-grade tumors had an OS at 5 years of 86.3% versus 72% in patients with high-grade tumors (p = 0.492) (Figure 2A). Patients with tumors <5 cm had an OS at 5 years of 87.7% versus 64.2% in patients with tumors >5 cm (p = 0.106) (Figure 2B). Pediatrics had an OS at 5 years of 80.8% versus 78.8% in AYA (p = 0.749) (Figure 2C). Use of chemotherapy and use of RT in the entire cohort were not associated with improved survival by the log-rank test.

On univariate analysis there was no difference in OS based on age group, gender, tumor grade, tumor size, use of RT, RT dose or use of chemotherapy (Table 2). Therefore, multivariate analysis was not performed.

**Table 2.** **Hazards ratio for death: univariate analysis.**

**Variable**	**Categories**	**Hazard ratio**	**95% CI**	**p-value**
Age group	Pediatrics	1	–	–

	AYA	1.200	0.391–3.680	0.748

Gender	Female	1	–	–

	Male	2.040	0.677–6.245	0.228

Grade	Low	1	–	–

	High	1.643	0.391–6.909	0.491

Tumor size	<5 cm	1	–	–

	≥5 cm	2.541	0.606–10.649	0.193

Radiation	No RT	1	–	–

	RT (any dose)	0.847	0.269–2.670	0.776

RT dose	No RT or RT <54 Gy	1	–	–

	RT ≥54 Gy	0.478	0.105–2.184	0.304

Chemotherapy	No chemotherapy	1	–	–

	Chemotherapy	2.516	0.845–7.490	0.099

AYA: Adolescent and young adult (age 15–39 years); RT: Radiation therapy.

**Figure 3.**
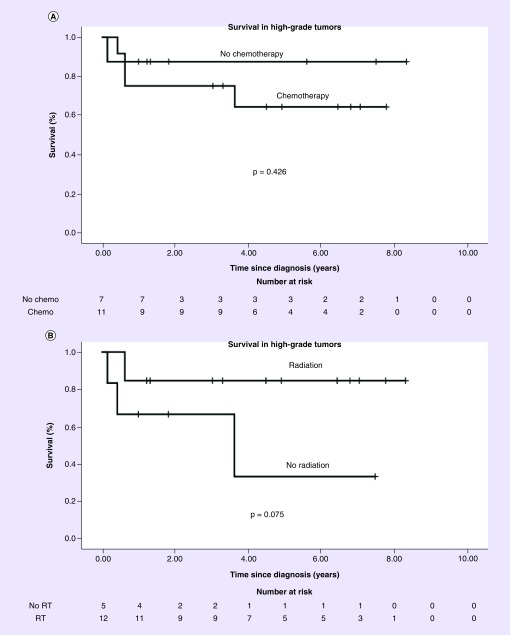
**Overall survival in high-grade tumors by treatment group.** **(A)** Overall survival in patients with high-grade tumors who received chemotherapy versus did not receive chemotherapy. **(B)** Overall survival in patients with high-grade tumors who received radiation therapy versus did not receive radiation therapy. RT: Radiotherapy.

On subgroup analysis of 20 patients with high-grade tumors, OS at 5 years was 87.5% in eight patients not receiving chemotherapy versus 64.3% in 12 patients receiving chemotherapy (p = 0.426) (Figure 3A). OS at 5 years was 33.3% in six patients not receiving RT versus 84.6% in 13 patients receiving RT (p = 0.075) (Figure 3B). Subgroup analysis of low-grade tumors was not performed, with only three patients with low-grade tumors receiving RT and one receiving chemotherapy.

## Discussion

The literature on astroblastoma is mostly limited to case studies, small case series, and literature reviews due to the rarity of the tumor. Ahmed *et al*. performed an analysis of the Surveillance, Epidemiology and End Results database on astroblastoma but lack critical information on tumor grade, radiation dose and technique and chemotherapy [3]. The current study presents demographic, tumor, treatment and outcomes data from 63 children and young adults with astroblastoma.

A total of 5 year OS of 79.5% in the current study is noteworthy given the unpredictable nature of astroblastoma in many case reports. The favorable survival in the current study is consistent with the favorable prognosis reported in other large cohorts of astroblastoma patients evaluated [2,3].

Data in the present study fail to identify an association between pathologist-assigned tumor grade and survival. The largest reports of astroblastoma patients in the literature do not evaluate tumor grade [2,3]. Thus, the current study represents the largest report of astroblastoma patients to be evaluated for an association between tumor grade and survival. With only 63 patients, the current study may be underpowered to detect such association. Additionally, more patients with high-grade tumors received RT, which likely reduces detection of any survival difference that exists. Larger studies with central pathologic review are necessary to define the association between pathohistological features and prognosis.

Patients with high-grade tumors were treated with chemotherapy at a much higher rate than patients with low-grade tumors. No benefit of chemotherapy was observed, even among patients with high-grade tumors. This should be considered in multidisciplinary discussions of patients with astroblastoma. The paradigm for systemic therapy may shift as more is learned about the molecular profile of astroblastomas and targeted therapies such as BRAF inhibitors are considered [15].

Our study finds that patients with high-grade tumors are being treated with RT at a much higher rate than patients with low-grade tumors. Among patients with high-grade tumors, patients who did not receive RT had poor survival. The survival benefit of RT for high-grade astroblastoma was not statistically significant, but is likely underpowered, with only 19 patients evaluated. Our results suggest RT is likely beneficial for patients with high-grade astroblastoma. Limited information is provided by the NCDB on target volumes for RT. Although recurrence in astroblastoma is most often local, distant CNS metastases of astroblastomas have also been observed [7,10,14]. The appropriate radiation field to treat is thus an area requiring further investigation.

Strengths of the current study include its relatively large number of patients compared with other analyses of astroblastoma, as well as information regarding grade, chemotherapy and details of radiotherapy. However, there are several limitations. There was no central pathological review, and tumor grade was known for only 60% of patients. Information on extent of resection, which has known prognostic significance and may influence subsequent management, is missing in the majority of patients. Specific information on chemotherapy regimen is not included in the NCDB.

To the authors’ knowledge, the current paper is the largest group of astroblastoma patients to include information on tumor grade, chemotherapy and details of RT.

## Conclusion

While astroblastoma has previously been considered a highly erratic and aggressive tumor, patients with astroblastoma should be treated with curative intent, as the majority of patients become long-term survivors. Patients with high-grade astroblastoma who do not receive RT experience poor survival. Thus, RT following surgical resection is likely beneficial in patients with high-grade astroblastoma. The exact role of RT and chemotherapy following surgical resection require further investigation.

## Future perspective

Proton therapy is likely already being used in the treatment of astroblastoma and will take on an increased role in treatment of this brain tumor in coming years, particularly in the pediatric, adolescent and young adult population. Greater molecular profiling of astroblastoma may provide greater insight on the ontological origins of this tumor and open doors for targeted therapies in the treatment of astroblastoma.
